# Racial, Ethnic, and Socioeconomic Disparities in Web-Based Patient Portal Usage Among Kidney and Liver Transplant Recipients: Cross-Sectional Study

**DOI:** 10.2196/11864

**Published:** 2019-04-22

**Authors:** Joel Wedd, Mohua Basu, Laura M Curtis, Kayla Smith, Denise J Lo, Marina Serper, Michael S Wolf, Ruth Parker, Rachel E Patzer

**Affiliations:** 1 Department of Medicine Emory University Atlanta, GA United States; 2 Emory Transplant Center Atlanta, GA United States; 3 Department of Medicine Northwestern University Chicago, IL United States; 4 Department of Medicine University of Pennsylvania Perelman School of Medicine Philadelphia, PA United States

**Keywords:** kidney transplantation, liver transplantation, patient portal, patient engagement, healthcare disparities

## Abstract

**Background:**

Kidney and liver transplant recipients must manage a complex care regimen after kidney transplant. Although the use of Web-based patient portals is known to improve patient-provider communication and health outcomes in chronic disease populations by helping patients manage posttransplant care, disparities in access to and use of portals have been reported. Little is known about portal usage and disparities among kidney and liver transplant recipients.

**Objective:**

The aim of this study was to examine patient racial/ethnic, socioeconomic, and clinical characteristics associated with portal usage among kidney and liver transplant recipients.

**Methods:**

The study included all adult kidney and liver transplant recipients (n=710) at a large academic transplant center in the Southeastern United States between March 2014 and November 2016. Electronic medical record data were linked with Cerner portal usage data. Patient portal use was defined as any portal activity (vs no activity) recorded in the Cerner Web-based portal, including viewing of health records, lab results, medication lists, and the use of secure messaging. Multivariable log-binomial regression was used to determine the patient demographic, clinical, and socioeconomic characteristics associated with portal usage, stratified by organ.

**Results:**

Among 710 transplant recipients (n=455 kidney, n=255 liver), 55.4% (252/455) of kidney recipients and 48.2% (123/255) of liver recipients used the patient portal. Black patients were less likely to use the portal versus white patients among both kidney (57% black vs 74% white) and liver (28% black vs 55% white) transplant recipients. In adjusted multivariable analyses, kidney transplant recipients were more likely to use the portal if they had higher education; among liver recipients, patients who were white versus black and had higher education were more likely to use the portal.

**Conclusions:**

Despite studies showing that patient portals have the potential to benefit transplant recipients as a tool for health management, racial and socioeconomic disparities should be considered before widespread implementation. Transplant centers should include portal training and support to all patients to encourage use, given its potential to improve outcomes.

## Introduction

### Complexity of Transplant Care

Transplantation has improved health outcomes for patients with end-stage organ failure, doubling median survival rates compared with patients who remain on the waiting list for an organ (kidney 12.4 years vs 5.4 years; liver 11.6 years vs 3.1 years; heart 9.5 years vs 2.3 years; and lung 5.2 years vs 2.3 years) in the last 25 years [1-3]. Immunosuppressant therapy is essential to graft survival, and treatment regimens can be highly complex and a major burden for patients to manage [4]. Transplant recipients, for example, take an average of 11 medications per day and often experience frequent changes in their medication regimen [5,6]. In addition, because of the complex and multidisciplinary nature of their clinical management, transplant recipients are required to navigate complex systems of care by attending frequent clinical appointments, obtaining frequent laboratory and radiologic testing, and meeting with multiple medical specialists.

### Potential Impact of Internet Technologies Upon Transplant Care

Modern internet technologies have been used to help manage other complex conditions, including diabetes, HIV, asthma, depression, heart disease, tuberculosis, and end-stage renal disease, leading to improvement in overall health and self-care management [7-13]. These new technologies have increased patient empowerment, decreased office visit rates and phone calls to physicians, and improved adherence to treatment [14,15]. However, little is known about the use of modern Web-based technologies among the transplant recipient patient population. Studies have indicated that owing to the high prescription burden, transplant recipients are interested in Web-based applications to assist in their complex medication management [16]. For example, Browning et al reported that 78% of adult kidney recipients had a positive attitude toward the use of mobile health smartphone apps for medication management [17].

Web-based patient portal systems can enhance convenience, communications, and fidelity in patient care. Despite previous research showing positive patient attitudes toward health technology, it may not be adopted equitably by all patients, and patient portal use is likely decreased in patients of minority race, lower educational level, and older age [18-21]. For example, non-Hispanic whites, younger adults, and those with high levels of education are more likely to use patient portals for diabetes management compared with racial and ethnic minorities, older adults, and those with lower education [18]. Limited access to technology may be a contributor to this gap. Other studies, however, suggest that the observed disparities are less related to access but that a lack of familiarity with data retrieval (eg, electronic health literacy) helps explain the lower portal usage among these disparate groups [22]. Among hospitalized patients with low socioeconomic status, inadequate communication between patients and providers during discharge and posthospital transition periods has also been cited as a potential barrier to medication access and adherence [23]. A patient portal has the potential to overcome barriers in patient-provider communication, but little is known about potential disparities in portal usage among kidney and liver transplant recipients.

The aim of this study was to examine the patient demographic, clinical, and socioeconomic characteristics associated with kidney and liver transplant recipients’ usage of a patient portal among a diverse population transplanted at a large transplant center in the Southeastern United States. Results of this study may help transplant centers better understand which transplant patients use the portal as we seek to improve portal use as a means of improving patient-provider communications and health outcomes.

## Methods

### Study Design

In this cross-sectional study, we examined patient portal use among all adult patients (N=710) who received a single-organ kidney or liver transplant at a large transplant center in the Southeast between March 2014 and November 2016. Owing to significant clinical and programmatic differences between kidney and liver recipients, which may impact portal use, they were treated as 2 separate cohorts. The patient Web portal is part of the institutional electronic medical record (EMR) system and integrates clinical information into 1 accessible Web interface for patients. Patient information is summarized on the portal landing page, and tabs direct patients to specific functions such as health record overview, medication overview, appointment management, prescription renewal, and bill payment. Health records include laboratory results, which are updated within 36 hours, and radiology reports, which are updated within 7 days, after authorization and review by the ordering physician. The messaging function allows patients to communicate with providers and transplant nurse coordinators. Since the launch of the patient Web portal in March 2014, the patient portal was mentioned to kidney and liver candidates at evaluation for a transplant. At the time of evaluation for kidney candidates and before discharge after transplantation for both liver and kidney recipients, patients received instructions on how to access the portal and were informed that they would receive an email invitation prompting them to register for the portal.

### Data Sources

Kidney and liver transplant recipients were identified using the transplant center’s EMR data for demographic variables and linked with data from the national United Network for Organ Sharing (UNOS) database to collect additional demographic, clinical, and socioeconomic variables. All demographic, clinical, and socioeconomic variables were obtained from UNOS with the exception of marital status, which was obtained from the institution’s EMR. If data were missing from UNOS, they were extracted from the EMR if available. Patient zip code was linked to US Census American Community Survey 5-year data from 2014 to collect zip code–level data on poverty. In addition, patient data were linked to patient portal activity, which was documented in Web server logs as part of the institution’s EMR (Cerner Corp, North Kansas City, MO) and included each portal function performed with a timestamp.

### Study Variables

The main outcome was patient usage of the Web portal, defined as whether a patient had any recorded activity based on Cerner server logs at any point during the study period, which includes 6 months before transplant and 2 years after transplant.

Demographic variables included age at transplant, gender, race/ethnicity (white, black, Hispanic, or other), marital status (single vs married), patient’s highest level of education (categorized as grade and high school, some college, and college/graduate degree). Sociodemographic variables included insurance at the time of transplant (private vs public, which included Medicare and Medicaid), employment at wait-listing, percent of people living under the federal poverty line in the patient’s zip code (categorized as <10%, 10 to 15%, or ≥15%), and patient distance from transplant center (distance between zip codes). Clinical characteristics included disease etiology, length of stay during time of transplant (calculated by subtracting transplant date from discharge date), functional status at the time of transplant measured by Karnofsky Performance Score (categorized as < vs ≥80%, which was defined as “Normal activity with effort: some symptoms of disease”). For kidney transplant recipients, we collected donor type (deceased or living). For liver transplant recipients, measures of liver disease severity at the time of transplant such as the laboratory model for end-stage liver disease (MELD) score and presence of encephalopathy were also collected. In the liver transplant cohort, we combined Hispanic race/ethnicity with the *other* race/ethnicity category for higher predictive power. All variables were obtained from UNOS.

In addition, exploratory outcomes were collected, including patient use of specific portal functions over the study period, which were measured using Cerner server logs documenting whether a patient used the portal to make appointments; to view clinical documents, current medications, current allergies, immunization records, visit history, radiology results, or lab results; or to engage in clinical messaging with providers. All time stamps were standardized in reference to the transplant date (t=0). Portal use was measured as a rate: number of clicks in the portal per 100 patients between March 2014 and November 2016.

### Statistical Analyses

We compared patient characteristics of portal users versus nonusers by using chi-square tests for categorical variables and independent *t* tests or Wilcoxon Rank Sum tests for continuous variables. All variables that were significant in these tests were included in a multivariable model. Using backward selection, variables with a risk ratio of 1 in the multivariable model were excluded from the final model. For variables with >10% missing data, a missing category was included for analyses. In addition, descriptive statistics for frequency of portal use functions were calculated for kidney and liver patients.

## Results

### Characteristics of Patient Population

Patient characteristics of 710 kidney and liver transplant recipients included in the study population are shown in Tables 1 and 2, respectively, of which 64.1% (455/710) were kidney transplant recipients and 35.9% (255/710) were liver transplant recipients; results are stratified by portal usage. Portal usage was slightly higher in kidney transplant patients (55.4%, 252/455) compared with the liver cohort (48.2%, 123/255). The median age of kidney transplant recipients was 49, and the majority of patients were black (56.9%, 259/455) followed by white (33.9%, 154/455), Hispanic (4.4%, 20/455), and other race/ethnicity (4.8%, 22/455). More than half of the kidney recipients were male (55.2%, 251/455)), married (53.4%, 243/455) with the majority having public insurance (73.6%, 335/455), being unemployed at time of wait-listing (55.2%, 251/455), and receiving a deceased donor transplant (61.5%, 280/455). Among the liver transplant recipients, the median age was 53 and the majority of patients were male (59.6%, 152/255), white (71.8%, 183/255), followed by black (23.9%, 61/255), Hispanic (0.8%, 2/255), and other race/ethnicity (3.5%, 9/255). Most patients were married (65.5%, 167/255), had lower education (42.7%, 109/255 completed grade or high school only), and had private insurance (58.4%, 149/255).

**Table 1:** Characteristics of kidney transplant recipients between March 2014 and November 2016, by portal use.

Characteristics		Kidney			
		All (N=455)	User (N=252)	Nonuser (N=203)	*P* value
Age at transplant, median (IQR^a^)		49.1 (13.0)	48.1 (19.1)	50.3 (24.8)	.08
**Gender, n (%)**					>.99
	Male	251 (55.2)	139 (55.2)	112 (55.2)	—^b^
	Female	204 (44.8)	113 (44.8)	91 (44.3)	—
**Race/Ethnicity, n (%)**					<.001
	White	154 (33.9)	114 (45.2)	40 (19.7)	—
	Black	259 (56.9)	120 (47.6)	139 (68.5)	—
	Hispanic	20 (4.4)	5 (2.0)	15 (7.4)	—
	Other	22 (4.8)	13 (5.2)	9 (4.4)	—
**Marital Status, n (%)**					0.03
	Single	208 (45.7)	104 (41.3)	104 (51.2)	—
	Married	243 (53.4)	147 (58.3)	96 (47.3)	—
	Missing	4 (0.8)	1 (0.4)	3 (1.5)	—
**Education new, n (%)**					<.001
	Grade and high school	147 (32.3)	51 (20.2)	97 (47.8)	—
	Some college	152 (33.4)	86 (34.1)	66 (32.5)	—
	College/graduate degree	147 (32.3)	110 (43.7)	37 (18.2)	—
	Missing	8 (1.8)	5 (2.0)	3 (1.5)	—
**Insurance category, n (%)**					<.001
	Public	335 (73.6)	160 (75.4)	175 (86.2)	—
	Private	120 (26.4)	92 (36.5)	28 (13.8)	—
**Employment at listing, n (%)**					<.001
	Yes	179 (39.3)	130 (51.6)	49 (24.1)	—
	No	270 (59.3)	120 (47.6)	150 (73.9)	—
	Missing	6 (1.3)	2 (0.8)	4 (2.0)	—
**US Census poverty level by zip code, n (%)**					<.001
	>15% (poorest)	204 (44.8)	91 (36.1)	113 (55.7)	—
	10-15%	100 (22.0)	60 (23.8)	40 (19.7)	—
	<10% (wealthiest)	145 (31.9)	100 (39.7)	45 (22.2)	—
	Missing	6 (1.3)	1 (0.4)	5 (2.5)	—
Distance (miles), median (IQR)		25 (14-68)	25 (17-57)	26 (13-79)	.81
Days on waiting list, median (IQR)		671 (258-1413)	608 (231-1,348)	798 (305-1,464)	.06
Length of stay, median (IQR)		4 (3-5)	4 (3-5)	4 (4-6)	<.001
**Etiology (kidney), n (%)**					<.001
	Diabetes	86 (18.9)	43 (17.1)	43 (21.2)	—
	Hypertension	156 (34.3)	61 (24.2)	95 (46.8)	—
	Glomerulonephritis	117 (25.7)	83 (32.9)	34 (16.7)	—
	Other	96 (21.1)	65 (25.8)	31 (15.3)	—




**Functional status group, n (%)**					<.001
	<80%	192 (42.2)	82 (32.5)	110 (54.2)	—
	>80%	259 (56.9)	168 (66.6)	91 (44.8)	—
	Missing	4 (0.9)	2 (0.8)	2 (1.0)	—
**Donor type, n (%)**					<.001
	Deceased	280 (61.5)	125 (49.6)	155 (76.4)	—
	Living	175 (38.5)	127 (50.4)	48 (23.6)	—

^a^IQR: interquartile range.

^b^Missing data or not applicable.

### Patient Portal Use Among Kidney Transplant Recipients

About half (55.4%, 252/455) of kidney transplant recipients were portal users (Table 1). Portal usage was the same among males and females (55.2%, 139/252 and 112/203), but portal users were slightly younger than nonusers (median: 49 vs 51; *P*=.08). Only 46.3% (120/259) of black patients and 25% (5/20) of Hispanic patients used the portal compared with 74.0% (114/154) of white patients (*P*<.001). Married versus single patients (60.5%, 147/243 vs 50.0%, 104/208; *P*=.03), patients with college or graduate school education versus grade/high school education (74.8%, 110/147 vs 34.7%, 51/147; *P*<.001), patients with private versus public insurance (76.7%, 92/120 vs 47.8%, 160/335; *P*<.001), patients employed versus unemployed (72.6%, 130/179 vs 44.4%, 120/270; *P*<.001), and the highest zip code poverty–level group versus the lowest zip code poverty–level group (69.0%, 100/145 vs 44.6%, 91/204; *P*<.001) were more likely to use the portal. Portal users had shorter times on the kidney transplant waiting list compared with nonportal users (608 vs 798 days; *P*=.06). Patients able to perform normal activity upon discharge used the portal more often compared with patients who were not able to perform normal activity (64.9%, 168/259 vs 42.7%, 82/192; *P*<.001). Patients with a disease etiology of glomerulonephritis (70.9%, 83/117 used portal) or *other* cause (68%, 65/96 used portal) were more likely to be portal users compared with patients with diabetes (50%, 43/86) or hypertension (39%; *P*<.001). Patients with higher (≥80%) versus lower (<80%) functional status (64.9% (168/259) vs 42.7% (82/192); *P*<.001) and patients with a living versus deceased donor transplant (72.5%, 127/175 vs 44.6%, 125/280; *P*<.001) were also more likely to be portal users.

In multivariable adjusted log-binomial regression models, the following variables were included in the final model: race/ethnicity, education, employment, insurance, zip code poverty level, marital status, etiology, functional status, and donor type. Once adjusting for these covariates, only the education level remained significant (Table 3). Transplant recipients with a college or graduate degree were more likely to use the patient portal than patients who finished grade and high school only (adjusted risk ratio [aRR] 1.16; 95% CI 1.01-1.32).

**Table 2:** Characteristics of liver transplant recipients between March 2014 and November 2016, by organ type and portal use.

Characteristics		Liver			
		All (N=255)	User (N=123)	Nonuser (N=132)	*P* value
Age at transplant, median (IQR^a^)		53.4 (11.5)	53.0 (11.7)	53.9 (11.3)	.64
**Gender, n (%)**					.27
	Male	152 (59.6)	69 (56.1)	83 (62.9)	—^b^
	Female	103 (40.4)	54 (43.9)	49 (37.1)	—
**Race/Ethnicity, n (%)**					.003
	White	183 (71.8)	101 (82.1)	82 (62.1)	—
	Black	61 (23.9)	17 (13.8)	44 (33.3)	—
	Hispanic	2 (0.8)	1 (0.8)	1 (0.8)	—
	Other	9 (3.5)	4 (3.3)	5 (3.8)	—
**Marital status, n (%)**					.91
	Single	88 (34.5)	42 (34.1)	46 (34.8)	—
	Married	167 (65.5)	81 (65.9)	86 (65.2)	—
	Missing	0 (0.0)	0 (0.0)	0 (0.0)	—
**Education new, n (%)**					<.001
	Grade and high school	109 (42.7)	40 (32.5)	69 (52.3)	—
	Some college	49 (19.2)	30 (24.4)	19 (14.4)	—
	College/graduate degree	51 (20.0)	36 (29.3)	15 (11.4)	—
	Missing	46 (18.0)	17 (13.8)	29 (22.0)	—
**Insurance category, n (%)**					.12
	Public	106 (41.6)	45 (36.6)	61 (46.2)	—
	Private	149 (58.4)	78 (63.4)	71 (53.8)	—
**Employment at listing, n (%)**					.02
	Yes	22 (8.6)	16 (13.0)	6 (4.5)	—
	No	221(86.7)	103 (83.7)	118 (89.4)	—
	Missing	12 (4.7)	4 (3.3)	8 (6.1)	—
**US Census poverty level by zip code, n (%)**					.69
	>15% (poorest)	101 (39.6)	45 (36.6)	56 (42.4)	—
	10-15%	67 (26.3)	34 (27.6)	33 (25.0)	—
	<10% (wealthiest)	85 (33.3)	42 (34.1)	43 (32.6)	—
	Missing	2 (0.8)	2 (1.6)	0 (0.0)	—
Distance (miles), median (IQR)		40 (21-87)	37 (21-78)	42 (20-99)	.73
Days on waiting list, median (IQR)		53 (7-128)	57 (13-137)	43 (5-122)	.11
Length of stay, median (IQR)		10 (8-18)	10 (7-18)	11 (8-19)	.21
MELD Score, mean (SD)		23.0 (9.4)	21.7 (9.3)	24.2 (9.4)	.03
**Encephalopathy at transplant, n (%)**					.37
	None	178 (69.8)	91 (74.0)	87 (65.9)	—
	2-Jan	67 (26.3)	28 (22.8)	39 (29.5)	—
	4-Mar	10 (3.9)	4 (3.3)	6 (4.5)	—




**Etiology (liver), n (%)**					.08
	Viral	91 (35.7)	37 (30.1)	54 (40.9)	—
	Alcohol	48 (18.8)	29 (23.6)	19 (14.4)	—
	Other	116 (45.5)	57 (46.3)	59 (44.7)	—
**Functional status group, n (%)**					.38
	<80%	87 (34.1)	39 (31.7)	48 (36.3)	—
	>80%	162 (63.5)	82 (66.6)	80 (60.6)	—
	Missing	6 (2.4)	2 (1.6)	4 (3.1)	—

^a^IQR: interquartile range.

^b^Missing data or not applicable.

**Table 3:** Risk ratios for portal user versus nonusers among kidney transplant recipients between March 2014 and November 2016.

Characteristics		Multivariable model, adjusted risk ratio (95% CI)	*P* value
**Race/Ethnicity**			
	White	Reference^a^	—^b^
	Black	0.96 (0.84-1.09)	.49
	Hispanic	0.83 (0.62-1.12)	.22
	Other	0.92 (0.75-1.13)	.43
Married versus single		1.02 (0.93-1.12)	.63
**Education level**			.48
	Grade school and high school	Reference	—
	Some college	1.11 (0.98-1.26)	.09
	College/graduate degree	1.16 (1.01-1.32)	.03
Employed versus unemployed		1.07 (0.96-1.19)	.23
Private versus public insurance		1.02 (0.91-1.15)	.69
**US Census poverty level by zip code**			
	>15% (poorest)	Reference	—
	10%-15 %	1.08 (0.95-1.22)	.23
	<10% (wealthiest)	1.07 (0.95-1.20)	.24
**Etiology**			
	Diabetes	1.04 (0.90-1.21)	.60
	Hypertension	Reference	—
	Glomerulonephritis	1.12 (0.98-1.27)	.09
	Other	1.10 (0.95-1.27)	.19
>80% versus <80% function		1.06 (0.95-1.17)	.29
Living versus deceased donor		1.06 (0.94-1.19)	.34

^a^Reference: Comparison group to which all others within characteristic are compared with.

^b^Missing data or not applicable.

### Patient Portal Use Among Liver Transplant Recipients

About half (48.2%, 123/255) of liver transplant recipients were portal users, with portal use being higher among females versus males (52.4%, 54/103 vs 45.4%, 69/152; *P*=.27) and white versus black patients (55.2%, 101/183 vs 27.9%, 17/61; *P*=.27; *P*=.003) (Table 2). Patients with college or graduate school education versus those who completed grade/high school education only (71%, 36/51 vs 36.7% 40/109; *P*<.001), patients with private versus public insurance (52.3%, 78/149 vs 42.5%, 45/106; *P*=.12), and patients employed versus those unemployed (73%, 16/22 vs 46.6% 103/221; *P*=.02) were more likely to use the portal. Patients who used the portal had longer times on the liver transplant waiting list compared with nonportal users (57 versus 43 days; *P*=.11) and lower MELD scores compared with nonportal users (21.7 versus 24.2; *P*=.03). Patients with an alcohol-related disease etiology (60%, 29/48) were more likely to use the portal compared with patients with a viral cause (41%, 37/91) or cause categorized as other (49.1%, 57/116; *P*=.08). Age, marital status, zip code poverty level, and distance were similar among portal and nonportal users among this cohort.

In multivariable adjusted binomial regression models, gender, race/ethnicity, marital status, education, employment, insurance, etiology, and MELD score were included in the final model (Table 4). In the final adjusted model, only race/ethnicity and education level remained significant. Black patients were significantly less likely than white patients to utilize the portal (adjusted RR 0.65; 95% CI 0.46-0.92). Transplant recipients with some college education or with a college or graduate degree were more likely to use the portal compared with those with a grade or high school education (some college versus grade/high school adjusted RR 1.36; 95% CI 1.01-1.84; college/graduate degree versus grade/high school adjusted RR 1.46; 95% CI 1.08-1.98; Table 4).

**Table 4:** Risk ratios for portal user versus nonusers among liver transplant recipients between March 2014 and November 2016.

Comparison groups		Multivariable model, adjusted risk ratio (95% CI)	*P* value
Female versus male		1.03 (0.83-1.29)	.76
**Race/Ethnicity**			
	White	Reference^a^	—^b^
	Black versus white	0.65 (0.46-0.92)	.01
	Other versus white	0.84 (0.53-1.32)	.44
**Education level**			
	Grade and high school	Reference	—
	Some college versus grade and high school	1.36 (1.01-1.84)	.04
	College/graduate degree versus grade and high school	1.46 (1.08-1.98)	.01
	Missing versus grade and high school	0.94 (0.64-1.38)	.76
Employed versus unemployed		1.11 (0.81-1.53)	.50
Private versus public insurance		1.03 (0.80-1.33)	.83
**Etiology**			
	Viral	0.84 (0.64-1.11)	.22
	Alcohol	1.14 (0.86-1.50)	.36
	Other	Reference	—
Model for end-stage liver disease score		0.99 (0.97-1.00)	.08

^a^Reference: Comparison group to which all others within characteristic are compared with.

^b^Missing data or not applicable.

### Portal Function Usage

**Figure 1:**
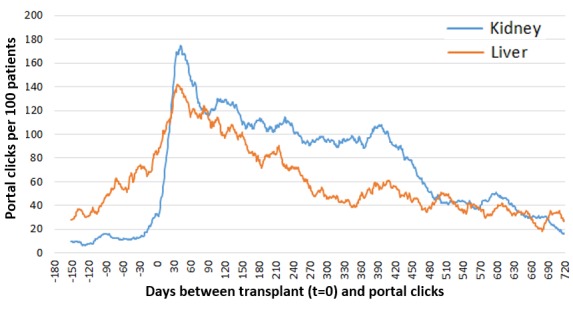
Overall portal activity among kidney (n=252) and liver (n=123) transplant recipients who registered for the patient portal between March 2014 and November 2016, 6 months before transplant to 2 years after transplant. Across the entire study period, portal functions were used at different rates among both.

In exploratory analyses among portal users, we found that the frequency of overall portal activity among both kidney and liver transplant recipients was similar; however, before transplantation, liver transplant recipients used the portal more frequently (Figure 1). Among both kidney and liver cohorts, there was an increase in recorded portal activity from 6 months (–150) before the time of transplant (t=0; approximately 180 clicks/100 kidney patients and approximately 140 clicks/100 liver patients), which gradually started declining 45 days after transplant. As portal activity gradually increased before transplantation in liver recipients, a more dramatic increase in portal clicks posttransplant was recorded in kidney recipients with more sustained use in the first 400 days of transplant (Figure 1).

Across the entire study period, portal functions were used at different rates among both kidney and liver transplant recipients (Table 5).

**Figure 2:**
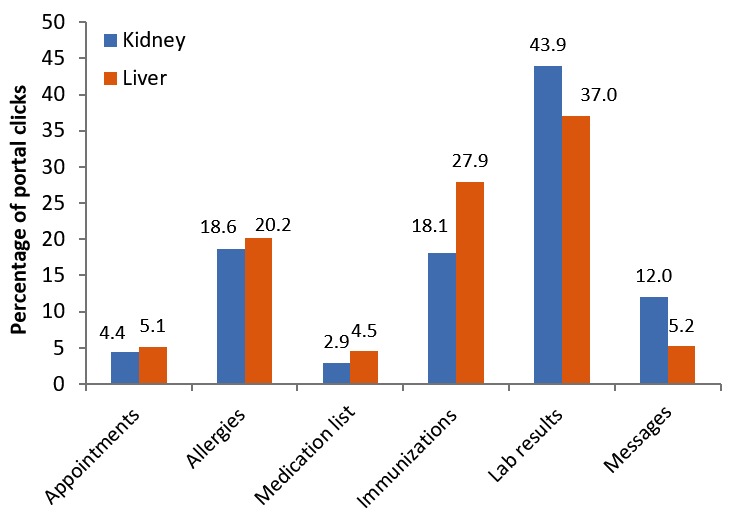
Portal function activity viewed among kidney (n=252) and liver (n=123) recipients who registered for the patient portal between March 2014 and November 2016, 6 months before transplant to 2 years after transplant.

Viewing lab results was the most frequent function used over the study period for kidney and liver patients (43.9% and 37.0%, respectively), followed by viewing immunizations (18.1% and 27.9%, respectively), allergies (18.6% and 20.2%, respectively), and messaging (12.0% and 5.2%, respectively; Figure 2). Portal activity for the different viewing functions was similar among kidney and liver transplant recipients, although kidney recipients tended to have higher activity viewing messages compared with liver patients, and liver patients had higher activity viewing immunizations compared with kidney patients.

**Table 5:** Portal functions used among kidney (n=252) and liver (n=123) transplant recipients who registered for the patient portal between March 2014 and November 2016, 6 months before transplant to 2 years after transplant. Percentages do not sum up to 100% due to rounding errors.

Portal functions	Kidney, n (%)	Liver, n (%)
Appointments	11 (4.4)	6 (5.1)
Allergies	47 (18.6)	25 (20.2)
Medication list	7 (2.9)	6 (4.5)
Immunizations	46 (18.1)	34 (27.9)
Lab results	111 (43.9)	46 (37.0)
Messages	30 (12.0)	6 (5.2)

## Discussion

In this single center cross-sectional study among a diverse population of kidney and liver transplant recipients at a major transplant center in the Southeast, Web-based patient portal usage was relatively low. Only about half of the patients used a Web-based patient portal at least one time after receiving a transplant, though previous literature has shown that this patient population is generally receptive to technological tools to help manage their complex medication regimes [17]. This study is the first to examine both kidney and liver transplant patients’ interaction with a Web-based patient portal, specifically with regard to the influence of socioeconomic characteristics on portal usage. In addition, this study uses novel data sources, including Web server logs to verify actual portal usage.

We identified significant racial and socioeconomic disparities in Web-based patient portal usage. Among kidney transplant recipients, patients with lower education levels were significantly less likely to use the portal. Among liver transplant recipients, black versus white patients and patients with lower education levels were less likely to use the portal. These results suggest that as patient portals have the potential for improvement in self-care management, strategies need to be developed to improve usage among minority patients and patients with lower education levels.

Certain findings in the multivariable analysis are not as clearly related to socioeconomic factors, and as there is sparsity in the literature of portal usage in transplant recipients, we speculate upon possible explanations. Kidney recipients with hypertension and diabetes as the cause of kidney disease were less likely to use the portal compared with those with other etiologies. In addition, among kidney recipients, living donor type and shorter length of stay during transplant were associated with increased portal usage. Kidney recipients receiving living donor kidney grafts may be less sick at transplant compared with deceased donor recipients who have spent more time on dialysis and thus less likely to use the portal. Similarly, patients with a longer length of hospital stay may be more complicated. Thus, degree of illness and complicated medical comorbidities may be markers for decreased portal use. As potential kidney recipients experience difficulty directly asking others to donate, kidney recipients with available living donors may have a more complete and comfortable social support network [24], which may also result in greater use of the patient portal by multiple family members. Liver recipients with higher laboratory MELD scores at transplant were also less likely to use the portal, again suggesting that the degree of illness at the time of transplant is associated with portal usage. Alternatively, high MELD patients are often acutely ill and newer to the system, limiting their familiarity with the portal. These findings suggest that patients who are more ill at the time of transplant may need to have transplant center staff reintroduce Web portals and their potential usefulness during subsequent appointments after transplant. In addition, patients who are more ill may benefit from more caregiver involvement.

Other studies have identified disparities in Web-based patient portal usage among patients with other chronic conditions [18,20,25-28]. For example, Goel et al reported a large racial/ethnic disparity in enrollment of their patient portal (Northwestern Medical Faculty Foundation) among a general internal medicine clinic population [20]. Roblin et al examined portal usage among adults with diabetes, adults with elevated lipids but no history of advanced coronary artery disease, and *low risk* adults in the Kaiser Permanente Georgia population, and found a similar distribution of racial and socioeconomic characteristics in portal usage, with socioeconomic characteristics not accounting for the disparities in usage by race/ethnicity [25]. In a separate study of adult diabetic patients, black and Hispanic patients had the highest odds of never logging on to the patient portal compared with non-Hispanic white patients [18]. Similar to our study, this study and others regarding patients with other conditions have shown that patients with lower socioeconomic status are less likely to access electronic health information [26].

Overall, the portal was used most within the first 30 days of transplant, but portal use declined after that time. Viewing lab results in the portal was the most used function by both kidney and liver patients. Portal activity was higher among patients awaiting liver transplant compared with those awaiting kidney transplant; however, after transplant, kidney recipients’ use increased above liver recipients’ use and was steady through, approximately, the next 500 days posttransplant. The reasons and implications of this pattern are unclear. Variations in clinic protocols between liver and kidney candidates before transplant may explain increased use in the liver recipients because pretransplant clinic interaction tends to be more rigorous for liver patients whereas kidney patients are often primarily managed by their preexisting nephrologist before transplant. Conversely, introduction to the patient portal included an instructional packet for kidney candidates but did not do so for liver candidates. However, this study does not explain the greater portal use by kidney patients after transplant. Use of the medication list function was low for both groups of recipients. Although the low usage may be related to center-specific protocols using alternative methods for medication reconciliation and communication, supporting the patient use of this function could translate to more accurate chart records of medications and could be the focus of future portal-based interventional studies.

Although internet access has been shown to be a contributing factor to racial disparities in portal usage, these disparities have decreased in the past decade; the proportion of whites, blacks, and Hispanics using the internet is 88%, 85%, and 88%, respectively [29]. Socioeconomic disparities are associated with health literacy challenges, so limited health literacy may both explain disparities in portal use but also help to identify individuals who could benefit most from portal-based initiatives [30]. This study suggests that when designing and implementing portals for transplant candidates and recipients, centers should consider socioeconomic factors, such as lower education levels, that may limit exposure of the portal to its target user and increase the need for targeted outreach and education about use and benefit of the portal.

There are several limitations to our study. Regional racial and socioeconomic composition is a limiting factor to the generalizability of this study. Among kidney transplant recipients, the proportion of African-Americans was higher compared with the proportion nationally reported in the OPTN/SRTR 2015 Annual Kidney Data Report (57% study vs 28% national) [3]. However, similar to the national data, kidney recipients were mostly publicly insured (74% study vs 67% national). Among liver transplant recipients, compared with the national data, the percentage of whites is similar (71% national vs 72% study), but the percentage of blacks is more than double the national rate (10% national vs 24% study), and most patients were privately insured (54% national vs 58% study) [2]. Other limitations of our study include lack of patient-reported data on the acceptability of portal use, lack of prospective follow-up to ascertain associations between portal use and outcomes, and lack of granular data about other potential socioeconomic variables associated with portal use. Findings from this study support the need for prospective and interventional studies using the patient portal in transplant populations.

On the basis of findings from this study and the other studies previously described, disparities in use of Web-based technologies seem to be universal among various health conditions, software systems, and US regions, implying a developmental flaw. Patient portals, as tools for health management, have the potential to benefit transplant recipients. However, racial and socioeconomic disparities should be considered during the development and implementation of any digital tool, including the development of innovative strategies to increase exposure and acceptance to a patient portal.

## Abbreviations

EMR: electronic medical record
MELD: model for end-stage liver disease
NIH: National Institutes of Health
RR: risk ratio
UNOS: United Network for Organ Sharing
